# Wnt inhibition alleviates resistance to immune checkpoint blockade in glioblastoma

**DOI:** 10.21203/rs.3.rs-3707472/v1

**Published:** 2023-12-26

**Authors:** Rakesh Jain, Shanmugarajan Krishnan, Somin Lee, Zohreh Amoozgar, Sonu Subudhi, Ashwin Kumar, Jessica Posada, Neal Lindeman, Pinji Lei, Mark Duquette, Sylvie Roberge, Peigen Huang, Patrik Andersson, Meenal Datta, Lance Munn, Dai Fukumura

**Affiliations:** MGH; MGH; MGH; MGH; Massachusetts General Hospital; MGH; MGH; Brigham and Women’s Hospital; MGH; MGH; MGH; MGH; MGH; MGH; MGH; Massachusetts General Hospital

## Abstract

Wnt signaling plays a critical role in the progression and treatment outcome of glioblastoma (GBM). Here, we identified WNT7b as a heretofore unknown mechanism of resistance to immune checkpoint inhibition (αPD1) in GBM patients and murine models. Acquired resistance to αPD1 was found to be associated with the upregulation of Wnt7b and β-catenin protein levels in GBM in patients and in a clinically relevant, stem-rich GBM model. Combining the porcupine inhibitor WNT974 with αPD1 prolonged the survival of GBM-bearing mice. However, this combination had a dichotomous response, with a subset of tumors showing refractoriness. WNT974 and αPD1 expanded a subset of DC3-like dendritic cells (DCs) and decreased the granulocytic myeloid-derived suppressor cells (gMDSCs) in the tumor microenvironment (TME). By contrast, monocytic MDSCs (mMDSCs) increased, while T-cell infiltration remained unchanged, suggesting potential TME-mediated resistance. Our preclinical findings warrant the testing of Wnt7b/β-catenin combined with αPD1 in GBM patients with elevated Wnt7b/β-catenin signaling.

## One Sentence Summary

Targeting Wnt overcomes resistance to αPD1 in glioblastoma.

## Introduction

Glioblastoma (GBM) is a fatal malignancy, with a median survival of less than 2 years with current treatments. Immune checkpoint blockers (ICBs) have provided durable responses in several malignancies but have failed in *all* Phase III trials in recurrent and newly diagnosed GBM [^1, 2^, NCT02617589] and in a randomized phase II trial in combination with bevacizumab ^3^. In the neoadjuvant setting, anti-programmed cell death I (αPD1) has shown benefit in a minor subset of patients in phase I/II trials ^4, 5^. Radiographic and clinical responses with ICBs in < 2% of GBMs with mismatch repair (MMR) deficiency or POLE deficiency have been reported ^6, 7^. This limited efficacy of ICBs is due to multiple factors including i) ICB-induced increase in brain edema that requires the use of immunosuppressive steroids ^8^, ii) poor infiltration and dysfunction of T cells in GBM ^9, 10, 11^, and iii) widespread immunosuppression in the GBM TME caused by infiltration of myeloid-derived cells from the bone marrow, and regulatory T cells (Treg cells) ^12, 13, 14^. Therefore, new therapeutic strategies are urgently needed to improve immunotherapy outcomes for GBM patients. The *Wnt* pathway is a key regulator of neural stem cells in embryonic development and adult neurogenesis ^15^. *Wnt* signaling is dysregulated in GBM and fuels GBM progression due to its role in proliferation, stemness and epithelial-mesenchymal transition (EMT) ^16, 17, 18, 19, 20, 21^. Hypermethylation of *Wnt* signaling repressors is observed in about 40–50% of GBM patients ^22^. Downregulation of Wnt inhibitory factor *(WIF)-1* in 75% of GBM indicates frequent involvement of aberrant *Wnt* signaling and may render GBM sensitive to inhibitors of *Wnt* signaling ^23, 24^. Here, we demonstrate that Wnt signaling is associated with resistance to αPD1 therapy in GBM patients. Targeting Wnt signaling using a porcupine inhibitor WNT974 that is in Phase I/II clinical trials for non-CNS tumors ^25^ alleviated immune suppression in a stem cell rich, αPD1-resistant GBM model in mice and improved their survival in combination with αPD1 treatment.

## Results

### Wnt7b/β-catenin elevation marks inherent and acquired resistance to αPD1 therapy.

**Figure 1:**
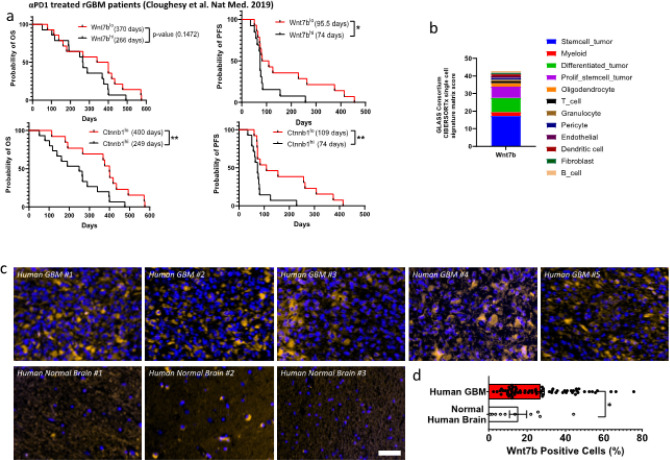
Wnt7b/β-catenin elevation associates with inherent and acquired resistance to αPD1 therapy. a) Overall survival (OS) and Progression free survival (PFS) in days for 28 patients was obtained from ^4^ GBM patient dataset and plotted based on their Wnt expression and Ctnnb1 expression. The expression above the median was considered ‘hi’ and below was considered ‘lo’. *p value<0.05, **p<0.01, Log Rank (Mantel-Cox) test. b) Expression of Wnt7b obtained by deconvolution of the GLASS gene expression dataset ^27^ by applying CIBERSORTx ^28^ using reference cell-state signatures derived from 55, 284 single-transcriptomes from 11 adult patients spanning glioma subtypes and time points ^29^. c) GBM patient tumor tissues were stained for Wnt7b protein using IF. Images of 5 representative patients from a total of 70 patients and 3 representative normal cerebrum tissues from a total of 10 normal tissues are shown. DAPI stain is shown in blue and anti-Wnt7b positive signaling is shown in orange color. Scale bar, 50 μM. d) Quantification of Wnt7b positive cells among total cells presented in each patient tissue. *p<0.05, Two-tailed, Unpaired student’s t-test. (n = 70 for GBM patient sample and n = 10 for normal brain tissue).

αPD1 refractory melanoma and hepatocellular carcinoma (HCC) exhibit elevated Wnt7b/β-catenin ^26^. To understand if this pathway is dysregulated in αPD1-treated GBM patients, we performed an analysis of a publicly available RNA-seq dataset (4). Almost all patients (28 out of 29) across two cohorts, either receiving neoadjuvant (N) + adjuvant (A) or adjuvant pembrolizumab only, had detectable levels of *Wnt7b* and *Ctnnb1* (**fig S1a**). Interestingly, *Wnt7b* was significantly elevated, and *Ctnnb1* trending towards higher expression in the cohort of patients receiving adjuvant pembrolizumab only (**fig S1a-b**). We then assessed whether this high *Wnt7b* and *Ctnnb1* expression post-αPD1 treatment was associated with a poor outcome. Using the median *Wnt7b* and *Ctnnb1* expression, the patients were divided into Wnt7b^lo^, *Wnt7b*^hi^, *Ctnnb1*^lo,^ and *Ctnnb1*^hi^ groups. Patients from the *Wnt7b*^hi^ group had a significantly worse PFS and the *Ctnnb1*^hi^ group had significantly worse PFS and OS as compared to the *Wnt7b*^lo^ and *Ctnnb1*^lo^ groups, respectively (Fig. 1a). To conclude, *Wnt7b* and *Ctnnb1* expression is elevated in αPD1-treated GBMs and associates with poor outcome.

To validate these findings, we next identified the major Wnt ligands expressed in GBMs in the Glioma Longitudinal Analysis (GLASS) Consortium gene expression dataset comprised 168 GBM patients (121 IDH wildtype and 40 IDH mutant) obtained from 37 hospitals worldwide ^27^. We used CIBERSORTx ^28^ to deconvolute the GLASS dataset and reference cell-state signatures derived from 55,284 single-transcriptomes from 11 adult patients spanning glioma subtypes and time points ^29^. This deconvolution revealed Wnt7b, Wnt4, and Wnt6 as the Wnt ligands expressed in 168 patients. Wnt7b was mostly expressed in stem-cell-rich tumors followed by differentiated tumors and proliferative stem cell tumors (Fig. 1b). Wnt4 and Wnt6 were mainly expressed in fibroblasts and pericytes (**fig. S1d**). Single cell RNA-seq data from 28 IDH-wildtype GBMs showed that *Wnt7b* is expressed in malignant cells, macrophages and CD8 T cells (**fig. S2a**) ^30^. Our TCGA analysis showed that the *Wnt* pathway genes are enriched in the Mesenchymal and Pro-neural GBM subtypes. Wnt7b staining in a tissue array comprising 70 GBM patient tumor tissues and 10 normal cerebrum tissues revealed that GBM tissues had elevated Wnt7b (Fig. 1c–d). In addition to the array, we stained for Wnt7b protein in 15 GBM biopsies from the Brigham and Women’s Hospital. All 15 patients showed elevated Wnt7b levels as compared to the 10 normal cerebrum tissues of the array. Representative images of 3 patients and the % Wnt7b + cells in whole tumors are shown in (**fig. S2b-c**). Collectively, these results indicate that Wnt7b/β-catenin elevation is associated with both inherent and acquired resistance to αPD1 therapy in GBM patients.

### Wnt7b is essential in the maintenance of GSC005 – a stem cell – rich murine GBM model.

**Figure 2:**
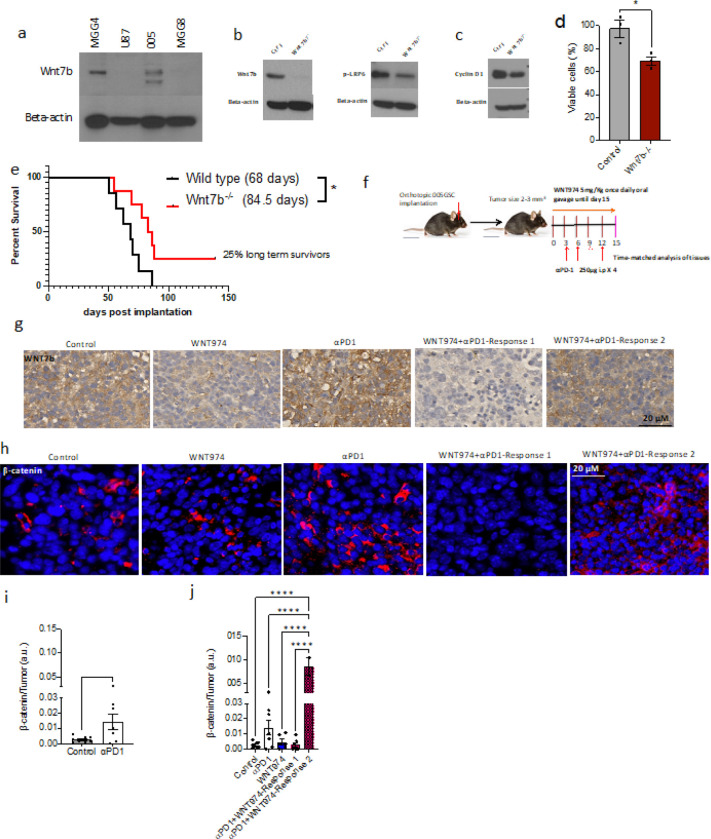
Wnt7b is essential in 005GSC maintenance, a murine model recapitulating stemness in GBM. a) Wnt7b protein levels were measured in lysates of patient derived GBM cell lines MGG4, U87 and MGG8. Murine 005GSC was used as a positive control. Western blot analysis of Wnt7b and pLRP6 (b) and Cyclin D1 (c) protein levels in wild type and Wnt7b^−/−^ cells. Beta-actin was used as a loading control. d) MTT assay performed at 120 h post seeding shows % viable cells. e) 5000 wild type or Wnt7b−/− cells were implanted in immune competent C57BL/6 mice (n=7 and 8, respectively) orthotopically, and survival was monitored. f) C57BL/6 mice were orthotopically implanted with 30,000 005-GSC-GFP. Mice were randomized at 3–4 mm^3^ into control, WNT974, αPD1, and WNT974+αPD1. Mice were treated with 250ug αPD1 or rat IgG i.p. once every 3 days for a total of 4 doses, WNT974 or equivalent methylcellulose was given by oral gavage at the dose of 5mg/kg once daily for 15 days and a time-matched histological analysis was performed. g) IHC (n=5–7 per group) and was performed for Wnt7b protein in a time-matched manner post treatment with WNT974, αPD1 and the combination. h) Immuno-fluorescence was performed on paraffin embedded sections for beta-catenin on the same tissues as (g). i-j) Beta-catenin staining was quantified as beta-catenin positive cells (nuclear and perinuclear) divided by the total number of tumor cells. DAPI shows the nuclear stain. Response 1) Tumors with decreased Wnt7b and β-catenin expression by WNT974+αPD1 as compared to the control. Response 2) Tumors with no alteration or increase of Wnt7b/beta-catenin expression by Wnt974+anti-PD-1 Median survival for each group is given in parentheses. *p<0.05, Two-tailed, Unpaired student’s t-test for MTT and Log-rank test for the survival study.

We next checked Wnt7b levels in human GBM cell lines: U87 (a widely used GBM cell line), and two cell lines generated from patients at MGH, MGG4 (stem-like) ^31^ and MGG8 (diffusely invasive) ^31^. Wnt7b was higher in MGG4 than MGG8 or U87. To capture this feature in a preclinical model, we used a stem-cell-derived murine GBM model 005GSC that harbors elevated expression of Wnt7b (Fig. 2a). To investigate the role of Wnt7b in tumor cell survival *in vitro* and *in vivo*, we performed CRISPR/Cas9-mediated deletion of *Wnt7b* and clonal selection. Western blot analysis revealed that as compared to the wild type 005GSC, Wnt7b protein was almost absent, and pLRP6-Ser1490 and Cyclin D1 (a known canonical Wnt target) were lower in the knockout clone (Fig. 2b, c). MTT assay performed at 120 h showed a 30% reduction in proliferation of the *Wnt7b*−/− clone (Fig. 2d).

We previously showed that 005GSC is an αPD1 resistant IDH wildtype murine model ^14^. Additionally, similar to human GBM, 005GSC is rich in stem cells, invasive and resistant to temozolomide (TMZ) ^32, 33^. These combined features make 005GSC a compelling model to study the role of Wnt7b in inherent and acquired resistance to αPD1. To this end, we implanted 5,000 wild type (n = 8) and 5,000 knockout cells (n = 7) orthotopically in mice. The median survival of the 005GSC^Wnt7bWt^-bearing mice was 68 days post implantation whereas that of the 005GSC^Wnt7b−/−^-bearing mice was significantly higher 84.5 days (p < 0.05; Fig. 2e). Moreover, 25% of the mice from the 005GSC^Wnt7b−/−^ group survived more than 135 days. (Fig. 2e). Supporting the role of Wnt pathway in acquired resistance to αPD1, our time-matched IHC analysis of 005GSC implanted mouse GBM tissues showed that αPD1 treatment led to elevated Wnt7b and β-catenin (nuclear and perinuclear) levels (Fig. 2f–j).

### Wnt inhibition reduces resistance to αPD1 in 005GSC GBMs.

**Figure 3:**
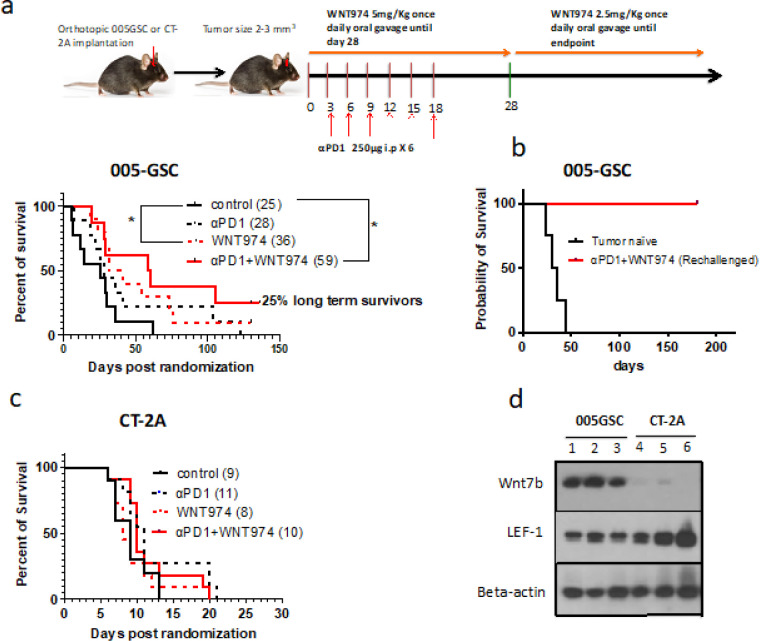
Wnt inhibition reduces resistance to αPD1 in 005GSC GBMs. 30,000 parental 005GSC-GFP (a) or 30,000 CT-2A-GFP (c) cells were orthotopically implanted and randomized into control, WNT974, αPD1 and WNT974+αPD1, and survival was monitored. The median survival for each arm is given in parentheses. b) 25% long-term survivors from experiments in (a) were rechallenged with 100,000 005GSC-GFP cells on their contralateral hemisphere. Percent survival is plotted. d) Wnt7b and Lef-1 protein levels in tissue lysates of 005GSC and CT-2A (n=3 samples per group). *p<0.05 using Log Rank test for survival study.

To assess whether Wnt-signaling contributes to αPD1 therapy resistance, we employed WNT-signaling, we used WNT974 - an inhibitor of porcupine (PORCN), an ER-resident O-acyltransferase that mediates Wnt palmitoylation, an essential step in Wnt biosynthesis ^34^. Firstly, we repeated our published findings in that GSC005 is resistant to αPD1 monotherapy (Fig. 3a). To investigated whether WNT974 in combination with αPD1 can improve the survival of tumor-bearing mice, we implanted 005GSC orthotopically, monitored tumor growth and randomized the mice into 4 groups – control (vehicle for WNT974 + IgG for αPD1), WNT974, αPD1 and WNT974 + αPD1. The median survival of the control group was 25 days. WNT974 improved the median survival significantly to 36 days, and WNT974 + αPD1 increased the survival to 59 days, with 2 out of 8 mice (25%) surviving more than 135 days (Fig. 3a). Of interest, tumors did not form in the long-term survivors challenged with 005GSC cells in the contralateral hemisphere suggesting that these mice had developed a memory response and were cured (Fig. 3b). By contrast, WNT974 alone or the combination with αPD1 did not produce any survival benefit in CT-2A model ^35^ (Fig. 3c). These differences in survival benefit are consistent with the low protein level of Wnt7b in CT-2A and high protein level of Wnt7b in 005GSC *in vivo* (n = 3) (Fig. 3d). Further, we observed that lymphoid enhancer binding factor (Lef-1), a downstream protein in the Wnt pathway was much higher in CT-2A as compared to 005GSC (Fig. 3d). Therefore, it is not expected that tumors with Lef-1 activation independently of canonical Wnt ligand signaling, such as CT-2A, would respond to WNT974.

Importantly, WNT974 has been shown to be minimally effective in tumors with downstream activation of lef-1: a mediator of Wnt signaling in EMT and brain metastasis, adenomatous polyposis coli (APC) or β-catenin mutations ^36, 37^, since these tumors are not driven by aberrant Wnt ligand expression. We profiled the 19 *Wnt* ligands in 005GSC and CT-2A *in vitro* and found that the former has a higher expression of *Wnt* ligands than the latter (**fig. S3a**). Among the Wnt ligands in 005GSC, *Wnt7b, Wnt7a, Wnt5a* had the highest expression. We then implanted both GBM models (n = 3 per group) in immune competent mice. The mRNA expression *in vivo* was also elevated similar to *in vitro* levels. Specifically, 005GSC had higher *Wnt5a, Wnt7a and Wnt7b* expression compared to CT-2A, emphasizing that CT-2A resistance to αPD1 is not related to the canonical Wnt pathway ^14, 38^. Note that *Wnt7b* expression is 4-fold higher than *Wnt7a* and *Wnt5a* in 005GSC (**fig. S3b: y-axis in log scale**). At the protein level, Wnt5a was not detectable in 005GSC.

Since WNT974 decreased the viable cells by 30% *in vitro* at 5μM and 10μM concentrations (**fig. S4a**), we used ApopTag to measure apoptosis in the WNT974, αPD1, and the combination groups but did not observe any difference as compared to control (**fig. S4b-c**). Thus, WNT974 modestly decreases proliferation of 005GSC, but has no effect on apoptosis.

### Response to therapy is caused by reduced levels of Wnt7b/β-catenin in 005GSC tumor cells and increased antigen presentation in the TME.

To dissect the mechanism of response, we interrogated whether WNT974 alone and/or in combination with αPD1 attenuated the Wnt pathway proteins and associated oncogenic signaling in tumor cells *in vivo*. We found that WNT974 monotherapy modestly decreased Wnt7b but did not affect beta-catenin levels in the tumor tissues (Fig. 2g–i). Intriguingly, WNT974 addition to αPD1 led to two strikingly different responses. In some tumors WNT974 attenuated αPD1-induced Wnt7b and beta-catenin (Response 1). In others, WNT974 + αPD1 did not change Wnt7b or β-catenin levels as compared to the control (Response 2) (Fig. 2g–j). On analyzing the phosphoproteome of the tumor tissues further, we identified that pro-oncogenic pathway proteins such as mTOR and MAPK/ERK were heavily phosphorylated at p-mTOR^Ser2448^ and pP44/42^Thr202/Tyr204^ sites, respectively, particularly in Response 2 (**fig. S5c**). Activation of these pathways has been implicated in activating β-catenin and downstream signaling. This is evident from the increased Lef-1 protein levels associated with Response 2 (**fig. S5c**).

**Figure 4:**
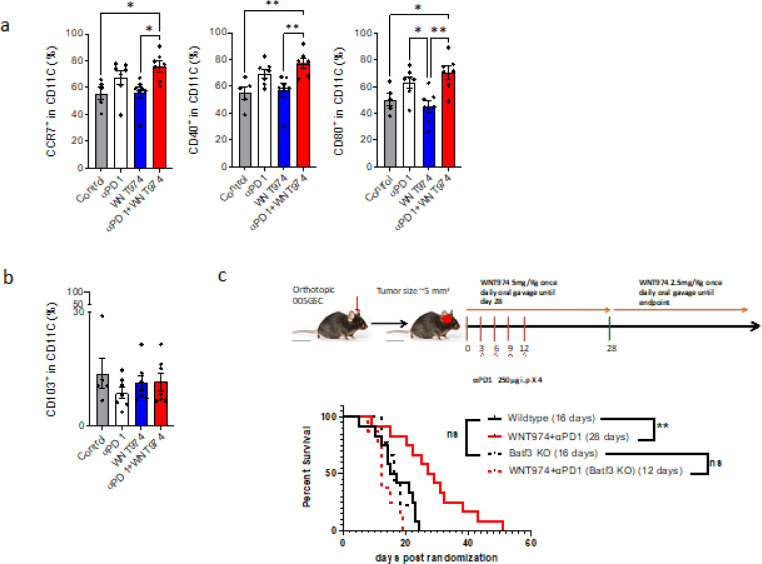
Response to therapy is due to increased antigen presentation in the TME. a) C57BL/6 mice were implanted with 30,000 005GSC followed by randomization at 3–4 mm^3^ into control, WNT974, αPD1 and WNT974+αPD1. On day 15, post-treatment tumor was harvested, digested, passed through a mesh, stained with antibodies indicated, made into single cells, and subjected to flow analysis. CD45+CD11C+ cells were identified as dendritic-like cells. Out of the CD45+CD11C+%, the CD40%, CCR7%, CD80% and b) CD103% were analyzed. * p<0.05, ** p<0.01. One-Way ANOVA followed by a test for multiple comparisons of means. c) 30,000 005GSC were implanted in *Batf3*^−/−^ mice with or without WNT974+αPD1. The survival benefit observed in wild-type mice was lost in *Batf3*^−/−^ mice. ** p<0.01 Log-rank test.

To elucidate the mechanisms that drives tumor cell death and elimination, we analyzed the tumor immune microenvironment (TIME) after treating mice with WNT974 and αPD1. We found that combined treatment with WNT974 and αPD1 maintained the overall hematopoietic immune cells marked by CD45. However, the DC3-like dendritic cells marked by expression of CCR7+, CD80+, or CD40 + increased (Fig. 4a) ^39, 40, 41, 42^. To ensure this phenotype is related to the function, we implanted 005GSCs in *Batf3*^−/−^mice that have reduced number of conventional dendritic cells (cDC1). The survival benefit observed in wild type mice by treatment with WNT974 + αPD1 was abrogated in *Batf3*^−/−^mice (Fig. 4c, Fig. 3a), suggesting that antigen presentation within the TME is a key driver of response.

### Reprogramming of MDSCs mediates resistance to WNT974 + αPD1

**Figure 5:**
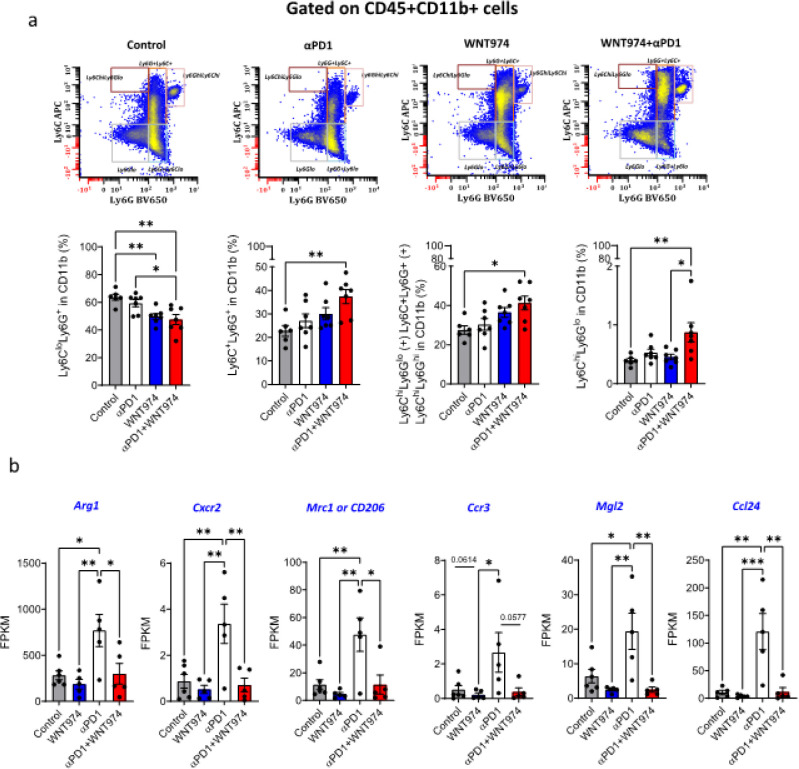
Reprogramming of MDSCs mediates resistance to WNT974+ αPD1. a)CD45+CD11b+ cells were gated for Ly6G and Ly6C populations. Ly6G+Ly6Clo% were identified as gMDSCs; Ly6G+Ly6C+ double positive populations were also analyzed. CD45+CD11b+Ly6C+ (includes Ly6GloLy6Chi, Ly6G+Ly6G+ and LyGhiLy6Chi cells) and CD45+CD11b+Ly6GloLy6Chi mMDSC populations were gated and graphed. Winlist was used to show the gating strategy in a representative bivariant plot. b) Bulk RNA-seq showing the Fragments per kilobase of transcript per million mapped reads of *Arginase1, Cxcr2, Mrc1, Mgl2* and *Ccl24* genes. * p<0.05, ** p<0.01, ***p<0.001. One-Way ANOVA. ** p<0.01, ****p<0.0001. One-Way ANOVA followed by a test for multiple comparisons of means.

Beyond DCs, we examined various subpopulations of CD45 + cells and found that Iba1 + cells, CD45 + CD11b + myeloid cells, CD45 + CD11b + Ly6G-Ly6C-F4/80 + tumor-associated macrophages, CD45 + CD11C + or CD45 + CD11C + CD103 + dendritic cells were not altered (**fig. S6a-c**, Fig. 4b). Within MDSCs, however CD45 + CD11b + Ly6G + Ly6Clo% gMDSCs decreased in the combination group as compared to control or αPD1 group (Fig. 5a). A double-positive population of Ly6G + Ly6C + cells increased in the combination group as compared to the control group as well as the αPD1 group (Fig. 5a). By investigating the potential players in mediating the reduction in gMDSCs via bulk RNA-seq analysis of tumors, we found that αPD1 treatment (a) increased the expression of *Arginase1, Mrc1 or CD206, and Mgl2* -- genes associated with immune suppression of MDSCs and tumor-brain interfacial macrophages/microglia ^43, 44, 45^, (b) increased *Ccl24* and (c) led to a trend towards increased *Ccr3*, a receptor of *Ccl24* involved in eosinophil, T cell and neutrophil chemotaxis (Fig. 5b). WNT974 addition to αPD1 alleviated these αPD1-induced changes in expression. Given the role of the vascular cell adhesion molecule 1 (VCAM1) in neutrophil infiltration ^46, 47, 48^, we performed a Western blot for VCAM1 in tumor tissue lysates (n = 3 mice per group). We found that WNT974 decreased VCAM1 levels to a greater extent than αPD1. The combination of WNT974 and αPD1 showed a reduction comparable to that of WNT974 as compared to control mice, indicating that blocking Wnt signaling is the main reason for the decrease in VCAM1 (**fig. S7a).** This pattern is consistent with the effect of WNT974, αPD1 and the combination on gMDSCs (Fig. 5a).

Our time-matched flow analysis indicated that the CD45 + CD11b + Ly6C + cells (that includes Ly6GloLy6Chi, Ly6G + Ly6G + and LyGhiLy6Chi cells) increased in the WNT974 + αPD1 group as compared to the control (Fig. 5a). Further, after eliminating the Ly6G + and Ly6Ghi cells, a purer mMDSC population CD45 + CD11b + Ly6ChiLy6Glo% increased in the WNT974 + αPD1 group as compared to the control or the WNT974 monotherapy group (Fig. 5a). CD4 + and CD8 + T cell % did not change with treatment in the TME (**fig. S8**).

## Discussion

**Figure 6:**
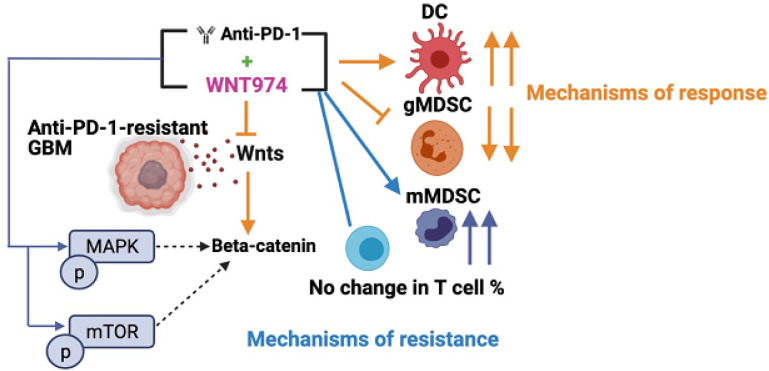
Overall findings. αPD1 resistant GBMs may respond to combinatorial therapy of Wnt inhibition and αPD1. Response to WNT974 and αPD1 may be mediated by an expansion of DC3-like DCs capable of antigen presentation and reduction in gMDSCs and associated immune suppression. This is accompanied by an increase in mMDSCs and insufficient T cell infiltration and function as modes of resistance. Additionally, resistance to WNT974 and αPD1 could be mediated by pro-oncogenic pathways such as mTOR and MAPK/ERK that could activate beta-catenin and sustain downstream Wnt signaling.

Wnt is essential for neurogenesis and regulation of the oligodendrocyte lineage. It is expressed in oligodendrogliomas and chemotherapy resistant GSC progenitor cells ^24^. This indicates that dysregulation of Wnt signaling in GBM is an early event in gliomagenesis but is maintained in WHO Grade IV, IDH-wild type tumors. We detected elevated Wnt7b protein levels in 100% of IDH-wild type human GBMs tested (**fig. S1b-c**), in the tissue array with 70 GBM tumors (Fig. 1c–d) and in IDH-wild type murine GBM 005GSC (Fig. 3d). CRISPR/Cas9-mediated knockout of Wnt7b in 005GSC implanted in immune competent mice prolonged survival (Fig. 2e). The combination of Wnt inhibition and αPD1/L-1 has been tested in extracranial tumors (**Supplementary Table 1**). However, the GBM TME is uniquely immune suppressive, and our mechanistic findings differ from previous extra-CNS studies (Fig. 6). Firstly, we identified that αPD1 treated GBM patients had high Wnt7b and Ctnnb1 expression and that this was associated with poor outcome in a public data set ^4^.

Previously it was shown that *Wnt*3a and EGFR mediate the binding of the beta-catenin/*Tcf/Lef* complex to *PDL1* and induce expression of *PDL1* in GBM ^49^. We observed that blocking PDL1 binding to PD-1 using αPD1 antibody can hyper-activate the Wnt pathway, resulting in escape (Fig. 2f–i). WNT974 addition to αPD1 inhibits the αPD1-induced Wnt7b and β-catenin levels in a subset of tumors (Fig. 2g–i).

One major reason for failure of therapies in GBM is intratumoral heterogeneity. Spatial profiling reveals regional heterogeneity in protein expression between distinct areas of the same GBM biopsy ^50^. Intratumoral hetereogneity correlates strongly with enrichment of stemness in multiple cancers including GBM ^51^ (Fig. 1b). Ineffective antitumor responses may result from high subclonality associated with an immune suppressive TME ^52^. Furthermore, due to their plasticity, GBMs may switch between cell states in response to therapy and escape treatment ^30, 53, 54^. A second interesting finding from our study is that WNT974 and αPD1 combination phosphorylated and activated pro-oncogenic pathways such as mTOR and MAPK/ERK in 005GSC (**fig. S5c**), which have been implicated in activation of β-catenin in a context dependent manner ^55, 56^. MAPK pathway alterations have been observed in the majority of ICB-responding GBMs and in one ICB non-responding GBM ^57^. A recent study showed that recurrent GBM patients who responded to αPD1 have increased phosphorylation of ERK1/2 and activation of a unique myeloid program with increased MHC-II expression. Further research in larger patient cohorts is warranted in this space ^58^. The MAPK pathway may increase IL-6 mRNA and protein ^59^ which in turn induces CCL2 to recruit mMDSCs. IL6 can also activate NFKappaB and STAT3 in infiltrating monocytes and GSCs via IL6R and cause immune suppression ^60^.

Third, WNT974 in combination with αPD1 improved the survival of αPD1 resistant, Wnt^hi^ 005GSC, whereas Wnt^lo^ CT-2A did not respond to either treatment (Fig. 3a,c). 25% of mice in the 005GSC study treated with the combination treated group were cured (Fig. 3b).

Fourth, we identified that a subset of DC3-like state of dendritic cells (CCR7+, CD40+, and CD80+) that mediate response to WNT974+αPD1 treatment in GBM (Fig. 4a). CD80, CCR7 and CD40 have been implicated in dendritic cell activation and maturation. CCR7+ DCs can migrate to lymph nodes to activate naïve T cells ^61, 62^. Our observation that CD103 is not associated with the DC3-like dendritic cell state is in line with other reports ^39–42^.

Although it has been reported that human DC3s develop via a specific pathway activated by GM-CSF, independent of cDC restricted and monocyte-restricted progenitors, they share features with conventional human DC subtypes. For example, CCR7 and CD40 upregulation has also been linked to cDC1 and cDC2 states in human breast tumors ^61^. Our results in GBM showing the involvement of CCR7+ and CD40+ DCs and a lack of response to WNT974+αPD1 in Batf3^−/−^ mice reveal the overlap between DC3-like cells and cDC1 ^63^.

We did not observe any changes in T cell infiltration with WNT974 and αPD1. It is plausible that the DC3-like DCs in GBM that are increased after WNT974 + αPD1 treatment acquire migratory and antigen cross-presentation capabilities, but may be limited by an mregDC program ^42^, or monocyte-like program that limits naïve T cell activation and proliferation in the draining lymph nodes. A recent intriguing study on neo-adjuvant αPD1 therapy in recurrent GBM patients showed an increase in 3 types of migratory DCs that are all CCR7+; however, the number of activated DCs was relatively low and did not lead to optimal antigen presentation and anti-tumor responses by T cells. Moreover, multiple immunosuppressive modules increased in the TME, and inhibitory checkpoints were upregulated on T cells ^64^.

Fifth, unlike previous studies (**Supplementary table 1**), we observed a decrease in gMDSC-associated immune suppression and an increase in mMDSCs with WNT974 and αPD1 (Fig. 5a,b). MDSCs consist of a heterogeneous population ^65^ and there are limited studies that define their role or target them in GBM ^66, 67^. Markers that distinguish their phenotype and function are limited. Published single cell studies have not revealed the full portrait of MDSCs ^12, 30, 68^ due in part to their lower number as compared to TAMs and to the functional and phenotypic similarity of gMDSCs to neutrophils. In our study the basal % of the Ly6G and Ly6C MDSC populations (Fig. 5a) match the % reported for 005GSC ^66, 69^ and have the potential to impact T cell activation and proliferation ^70^. We did not observe reduced gMDSCs in the TME of stem-like 005GSC, similar to what was reported for GL261 and SB28 cultured in serum conditions ^67^. Our results are in line with the findings that gMDSCs account for a significant fraction of MDSCs in the TME ^71, 72^. We used equal number of male and female mice in our MDSC profiling experiment and did not observe sexual dimorphism in MDSCs as previously reported ^67^.

Lastly, our finding that the combination of WNT974 and αPD1 decreases VCAM1 protein in GBM is novel. Reduction in VCAM1 levels with WNT974 alone, and the combination (**fig. S7a**) may be correlated with the reduction in gMDSC infiltration ^73, 74^. However, it is possible that reduction in VCAM1 could also impede T cells from infiltrating into the tumor. Additionally, among the 49 candidate ligand-receptor interactions associated with mesenchymal transition by longitudinal analysis and single-cell RNA-seq ^27^ identified VCAM1 on differentiated-like GBM tumors and myeloid cell expressing ITGB7 and ITGB1 as ligand-receptor pairs (**fig. S7b**). Therefore, our results on the decrease of VCAM1 in tumors are in line with the well known role of Wnt in epithelial-mesenchymal transtion.

Our study also has some limitations. i) Wnt inhibition may be overcome by activation of upstream genes, e.g., ASCL1 that are critical for the maintenance of GSCs ^75^ and other driver mutations. ii) The functionality of DCs and MDSCs identified in the study may need to be assessed using cross-presentation assays and T cell suppression assays, respectively, with WNT974 and αPD1 for which DCs and MDSCs could be isolated from lymph nodes and bone marrows respectively rather than the TME due to their low numbers in the TME. iii) The % of T cells infiltrated was not affected with WNT974 and αPD1. This does not mean that T cell states were not affected. Addressing this question requires an in-depth analysis of T cell activation, memory, and exhaustion states with WNT974 and αPD1.

In a fatal disease like GBM where treatment options are limited, the insights on the mechanisms of response and resistance to WNT974 and αPD1 from our study may help the development of personalized immune therapies in Wnt^hi^ GBMs. A phase I clinical trial of WNT974 in patients with advanced extracranial tumors showed that WNT974 was generally well tolerated ^76, 77^. Results from the phase I αPD1 antibody spartalizumab (PDR001)+WNT974 (NCT01351103) trial in non-CNS tumors show that such a combination increases T cell inflammation signature in some tumors, but decreases the signature in other tumors that were originally T cell inflamed, thus making them ‘cold’ ^36^. The resistance mechanisms that we have identified (increased phosphorylation of pro-tumorigenic pathway proteins, increased mMDSCs and insufficient T cell infiltration) may help clarify the results of this extra-CNS tumor trial.

## Materials and methods

### Cell lines, cell culture, and animals:

CT-2A is a mouse astrocytoma that was formed by chemical induction by methylcholanthrene in C57BL/6 mice; p53 wild type, PTEN deficient). CT-2A-GFP-Gluc was obtained from Dr. Thomas Seyfried of the Boston College. 005GSC-GFP was derived from GBM tumors induced by lentivirus transduction of H-Ras and Akt into the stem cell niche of Tp53+/− immunocompetent C57BL/6 mice. We received the 005GSC-GFP from Dr. Samuel Rabkins, MGH, Boston. Cells were cultured in serum free NeuroCult NS-A proliferation kit (Stemcell Technologies) in a humidified atmosphere of 5% CO2 and 95% air at 37°C. One day 10 post cranial window implantation, cells in 30,000 cells (unless specified) in 1ul of PBS were stereotactically implanted 2 mm left of the sagittal suture, 0.1 mm rostral of the bregma, and at a depth of 2 mm from the brain surface. 6–8-week-old C57BL/6 mice were used for *in vivo* experiments. IACUC and the DoD review committee approved all animal procedures.

### Cranial window implantation:

Once asleep after anesthesia, the head of an animal is fixed in a stereotactic apparatus. A 7mm circle is drilled over the frontal and parietal regions of the skull bilaterally. Using a bone micro drill with a burr-tip 1.4 mm in diameter, a first groove of 7 mm is made. This groove is made thinner by cautious drilling of the groove until the bone flap becomes loose. Cold saline is applied during the drilling process to avoid thermal injury of the cortical regions. Using a malis dissector, the bone flap is separated from the dura mater underneath. After removal of the bone flap, a wet (0.9% sodium chloride) piece of gelfoam is placed on the dura mater to keep it continuously moist. The dura and arachnoid membranes are removed completely from the surface of both hemispheres, avoiding any damage to the sagittal sinus. The window is sealed with a 7-mm cover glass that is glued to the bone with a mixture of histocompatible cyanoacrylate glue and fine acrylic powder. The animal is put on the warm plate, when waking up, it is put back in the cage and on the rack. It is monitored daily to ensure full recovery and given buprenorphine if needed.

### Tumor growth measurement:

005GSC tumor growth was monitored through plastic cranial windows using a small animal ultrasonography device (Vevo 2100, FujiFilm VisualSonics Inc.) ^83^. The average of the longest and shortest diameters was taken for calculation of tumor diameter. The growth of CT-2A-GFP-Gluc was monitored by serial blood Gluc measurements using a Promega Glomax luminometer and correlating it with tumor volume as measured by ultrasound ^84^.

### Statistical Analysis

Statistical analyses were performed using Graph Pad Prism Version 9.3.0. One-way ANOVA followed by Tukey’s multiple comparisons test was performed for all experiments except for survival studies where Log Rank test was used. p < 0.05 was considered significant. Two-tailed Unpaired Student’s T test was used for comparison between the two groups. The number of samples or animals ‘n’ is given in the Figure legends.

## Data Availability

All data associated with this study are included in the main manuscript or the Supplementary Materials.
